# Supplementary material for: Identification and functional analysis of non-coding regulatory small RNA FenSr3 in Bacillus amyloliquefaciens LPB-18
Source: PeerJ. 2023 May 15;11:e15236. doi: 10.7717/peerj.15236 (PMC10194069; doi:10.7717/peerj.15236)
Supplement: Supplemental Information 4 [file peerj-11-15236-s004.zip › KO/CK-vs-T1_map/map04064.html]

KEGG PATHWAY: NF-kappa B signaling pathway - Reference pathway


|  |  |
| --- | --- |
| **NF-kappa B signaling pathway - Reference pathway** |  |

[
Pathway menu
| Organism menu
| Pathway entry
| Show description
| User data mapping
]

Nuclear factor-kappa B (NF-kappa B) is the generic name of a family of transcription factors that function as dimers and regulate genes involved in immunity, inflammation and cell survival. There are several pathways leading to NF-kappa B-activation. The canonical pathway is induced by tumour necrosis factor-alpha (TNF-alpha), interleukin-1 (IL-1) or byproducts of bacterial and viral infections. This pathway relies on IKK- mediated IkappaB-alpha phosphorylation on Ser32 and 36, leading to its degradation, which allows the p50/p65 NF-kappa B dimer to enter the nucleus and activate gene transcription. Atypical pathways are IKK-independent and rely on phosphorylation of IkappaB-alpha on Tyr42 or on Ser residues in IkappaB-alpha PEST domain. The non-canonical pathway is triggered by particular members of the TNFR superfamily, such as lymphotoxin-beta (LT-beta) or BAFF. It involves NIK and IKK-alpha-mediated p100 phosphorylation and processing to p52, resulting in nuclear translocation of p52/RelB heterodimers.

|  |  |  |
| --- | --- | --- |
| Reference pathway | 184% 150% 122% 100% 82% 67% 55% | 图片下载 |
